# Incidência e Estudo Morfológico de Pontes Miocárdicas no Estado do Ceará: Um Estudo Cadavérico

**DOI:** 10.36660/abc.20220460

**Published:** 2023-06-28

**Authors:** Jalles Dantas de Lucena, Hudson Martins de Brito, João Victor Souza Sanders, Jonathan Barros Cavalcante, Michelly Carneiro Collyer, Cecília de Lima Leite, Helson Freitas da Silveira, Julio Cesar Campos Ferreira

**Affiliations:** 1 Centro Universitário Santa Maria Cajazeiras PB Brasil Centro Universitário Santa Maria, Cajazeiras, PB – Brasil; 2 Universidade Federal do Ceará Fortaleza CE Brasil Universidade Federal do Ceará, Fortaleza, CE – Brasil

**Keywords:** Anatomia, Ponte Miocárdica, Anormalidades Cardiovasculares, Incidência

## Abstract

**Fundamento:**

As pontes miocárdicas (PM) são anomalias anatômicas com possíveis repercussões clínicas, e, portanto, seu entendimento merece atenção.

**Objetivo:**

Para determinar a prevalência e caracterizar a PM em corações humanos do estado do Ceará. Métodos: Foram usados cinquenta corações de cadáveres humanos adultos da Faculdade de Medicina da Universidade Federal do Ceará, Brasil. Os corações foram dissecados para identificar PMs que passam sobre parte da artéria coronária. O segmento da artéria (proximal, médio e distal) com a ponte foi identificado. O diâmetro externo da artéria nos pontos proximal e distal da PM foi medido. O comprimento e a espessura da PM também foram medidos com um calibre eletrônico. O índice de massa muscular (IMM) da PM foi calculado como o produto do comprimento pela espessura expresso em milímetros. O nível de significância adotado para a análise estatística foi 5%.

**Resultados:**

A PM foi confirmada em 40% da amostra. Aproximadamente um terço da amostra tinha apenas 1 PM. A PM foi encontrada mais frequentemente sobre o ramo interventricular anterior da artéria coronária esquerda (59,25%, p = 0,02), e sua prevalência em outros ramos foi muito mais baixa (22,23%). Os segmentos das artérias mais afetados foram o superior (44,44%) e o médio (40,74%). O diâmetro médio das artérias proximais em relação à PM foi de 2,38 ± 0,97 mm (intervalo = 0,78 - 5,15 mm), e o diâmetro distal da PM foi de 1,71 ± 0,75 mm (intervalo = 0,42 - 3,58 mm). O comprimento foi medido como média = 8,55 ± 5,27 mm, e a espessura média foi de 0,89 ± 0,33 mm.

**Conclusão:**

A alta prevalência de PM tem mais probabilidade de afetar o sistema da artéria coronária esquerda com IMM maior do que outros ramos afetados.

## Introdução

A ponte miocárdica (PM) é uma anomalia arterial coronária congênita definida pelo envolvimento parcial de um ramo cardíaco arterial por fibras musculares miocárdicas, formando uma ponte muscular sobre os vasos envolvidos.^1 , 2^ A PM pode ser encontrada em qualquer artéria coronária epicárdica; entretanto, há uma prevalência maior ao longo do curso do ramo interventricular anterior (RIA) da artéria coronária esquerda (ACE) também chamada de artéria descendente anterior esquerda (ADAE).^3 , 4^ Essa variação anatômica é mais comum no segmento médio da artéria descendente anterior.^5^

Uma meta-análise recente demonstrou uma prevalência média da PM de 19%.^6^ Entretanto, dados sobre a frequência da PM geralmente são bastante variáveis na literatura, dependendo dos métodos usados para seu diagnóstico. Sobre isso, a prevalência tende a ser mais alta ao se considerar diagnósticos post-mortem.^2 , 7^ Atualmente, o uso de novas técnicas de exames complementares, tais como ultrassom intravascular e tomografia computadorizada cardíaca, aumentou a sensibilidade para detecção da PM, e permitiu uma caracterização morfológica e funcional desses achados anatômicos.^2 , 8^

A presença da PM é clinicamente relevante devido à sua associação com sintomas de angina, isquemia miocárdica, disfunção do ventrículo esquerdo, infarto agudo do miocárdio (IAM) ou mesmo morte súbita,^8 - 13^ e também é um fator de risco para doença arterial coronariana em várias situações clínicas.^8^ Os mecanismos fisiopatológicos de isquemia associados à PM ainda são controversos e mal entendidos devido às limitações da análise in vivo.^2 , 8^ Depois de o diagnóstico ser estabelecido, as medidas terapêuticas são essencialmente medicamentosas e tentam manter a frequência cardíaca em valores basais, considerados abordagens de primeira linha.^14^

O implante de stent sob a PM é, basicamente, uma negligência hoje em dia para uma variedade de complicações vitais agudas e de longo prazo, enquanto a descompressão cirúrgica é discutível e oferecida em poucos centros altamente especializados; portanto, não é viável para todos globalmente.^3^ Nessa perspectiva, é necessário investigar a prevalência da PM nas populações, por ser um achado relativamente comum e um importante fator de risco para patologias cardiovasculares com altas taxas de morbimortalidade, como arteriosclerose e IAM.^15 , 16^ Assim, este trabalho tem como objetivo estudar a prevalência da PM nos ramos das artérias coronárias em corações dissecados no estado do Ceará no nordeste do Brasil. Este trabalho também se propõe a analisar o comprimento e a espessura das pontes, e se há uma diferença no valor do índice de massa muscular da PM localizada acima do RIA e as pontes localizadas acima de outros ramos das artérias coronárias.

## Materiais e métodos

Foram obtidos cinquenta corações de cadáveres humanos adultos, dentro da conveniência do Departamento de Anatomia da Faculdade de Medicina da Universidade Federal do Ceará, Brasil. O número de corações não foi mais alto devido à escassez de espécimes disponíveis. Os corações foram preservados em glicerina até a análise. Informações, incluindo idade, gênero, etnia, histórico médico e causa da morte não puderam ser obtidas devido à ausência de registros.

Os espécimes foram selecionados a partir de uma amostragem intencional não probabilística de acordo com os seguintes critérios: coração com a presença do tronco arterial principal e seus ramos preservados tecido adiposo subepicárdico, músculo cardíaco preservado, garantindo a visualização das características morfológicas de cada espécime.

O epicárdio e a gordura epicárdica foram dissecados com cuidado. A partir daí, a origem e o trajeto das artérias coronárias e seus ramos importantes foram delineados com cuidado. Todos foram seguidos cuidadosamente para identificar qualquer PM passando sobre as artérias. Se a PM fosse detectada, o segmento da artéria (proximal, médio e distal) com a ponte era identificado. O diâmetro externo da artéria nos pontos proximal e distal imediatos da PM foi medido. Em seguida, o comprimento e a espessura da PM também foram medidos com a ajuda de um calibre eletrônico preciso (DIGIMESS®, São Paulo, Brasil) com precisão de 0,01 mm. O índice de massa muscular da PM foi calculado como o produto do comprimento pela espessura expresso em milímetros.

### Análise estatística

Os dados foram coletados em planilha de Excel e analisados estatisticamente usando o GraphPad Prism, versão 6.00, for Windows, California, EUA. Variáveis contínuas foram descritas por média e desvio padrão (DP) e foram analisadas como normalidade pelo teste de Shapiro-Wilk. As variáveis categóricas foram apresentadas em tabelas, com a frequência das variáveis estudadas. As comparações entre variáveis foram realizadas usando o teste t de Student não pareado. Valores em p <0,05 foram considerados estatisticamente significativos. O estudo anatômico foi realizado após a autorização do comitê de ética da Faculdade de Medicina da Universidade Federal do Ceará, Ceará, Brasil.

## Resultados

A presença de PM foi confirmada em 40% dos corações (n=20). Aproximadamente um terço da amostra (n=16, 32%) tinha apenas uma PM. nosso estudo também encontrou 4% (n=2) com duas PM (em artérias diferentes) e 2% (n=1) com três PM (2 PM acima do RIA) e quatro PM (2 PM acima do RIA) - Figura central .

**Figura Central:**
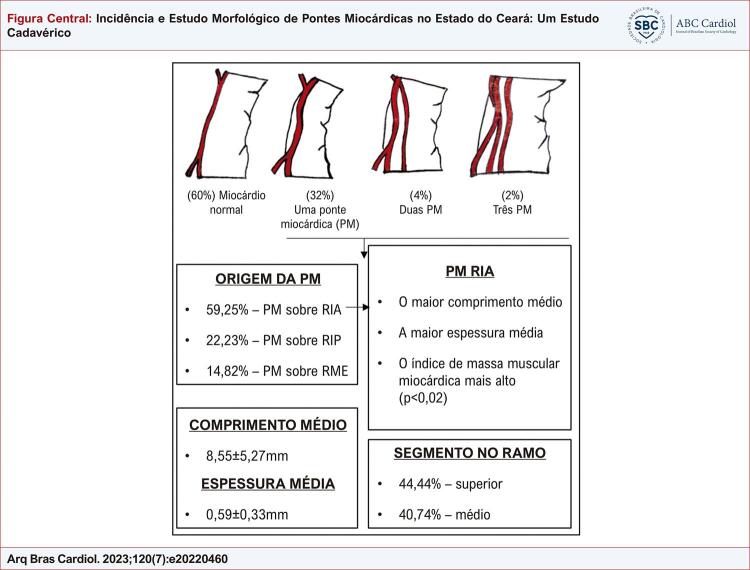
: Incidência e Estudo Morfológico de Pontes Miocárdicas no Estado do Ceará: Um Estudo Cadavérico

Principais achados deste artigo. PM: ponte miocárdica; RIA: ramo interventricular anterior; RIP: ramo interventricular posterior; RME: ramo marginal esquerdo.

Vinte e sete PM foram identificadas na amostra, mais comumente encontradas acima do RIA (n=16, 59,25%). A frequência de pontes acima de outros ramos foi bem menor, com 22,23% (n=6) acima do ramo interventricular posterior (RIP) e 14,82% (n=4) acima do ramo marginal esquerdo (RME) ( Tabela 1 , Figura central ).

**Tabela 1:** – Distribuição de pontes miocárdicas (n=27) acima das artérias nos corações (n=50)

Vaso sanguíneo	Corações com PM %	Número de PM
**RIA**	32,0	16 (59,25)
**RIP**	12,0	6 (22,23)
**RME**	8,0	4 (14,82)
**RVP**	2,0	1 (3,7)
**Total**	-	**27 (100,0)**

PM: ponte miocárdica; RIA: ramo interventricular anterior; RIP: ramo interventricular posterior; RME: ramo marginal esquerdo; RVP: ramo ventricular posterior.

As pontes miocárdicas estavam presentes principalmente nos segmentos superior (44,44%) e médio (40,74%) das artérias ( Figura central ). O diâmetro médio das artérias proximais em relação às pontes miocárdicas foi de 2,38 ± 0,97 mm (intervalo = 0,78 - 5,15 mm), e o diâmetro distal em relação às pontes miocárdicas foi de 1,71 ± 0,75 mm (intervalo = 0,42 - 3,58 mm).

O comprimento médio das pontes miocárdicas foi de 8,55 ± 5,27 mm (intervalo = 2,79 - 22,95 mm), enquanto a espessura média foi de 0,89 ± 0,33 mm (intervalo = 0,37-1,83 mm) - Figura central . Além disso, o valor do índice de massa muscular da ponte miocárdica (IMMPM) variou de um mínimo de 1,70 mm a um máximo de 28,69 mm e totalizou 8,19 ± 7,30 mm nas três artérias ( Tabela 2 ).

**Tabela 2:** – Aspectos morfológicos de pontes miocárdicas (N=27)

	Comprimento (mm)	Espessura (mm)	IMMPM
Média	8,55	0,89	8,19
Desvio padrão	5,27	0,33	7,30

IMMPM: índice de massa muscular da ponte miocárdica (comprimento x espessura).

**Figura 1:**
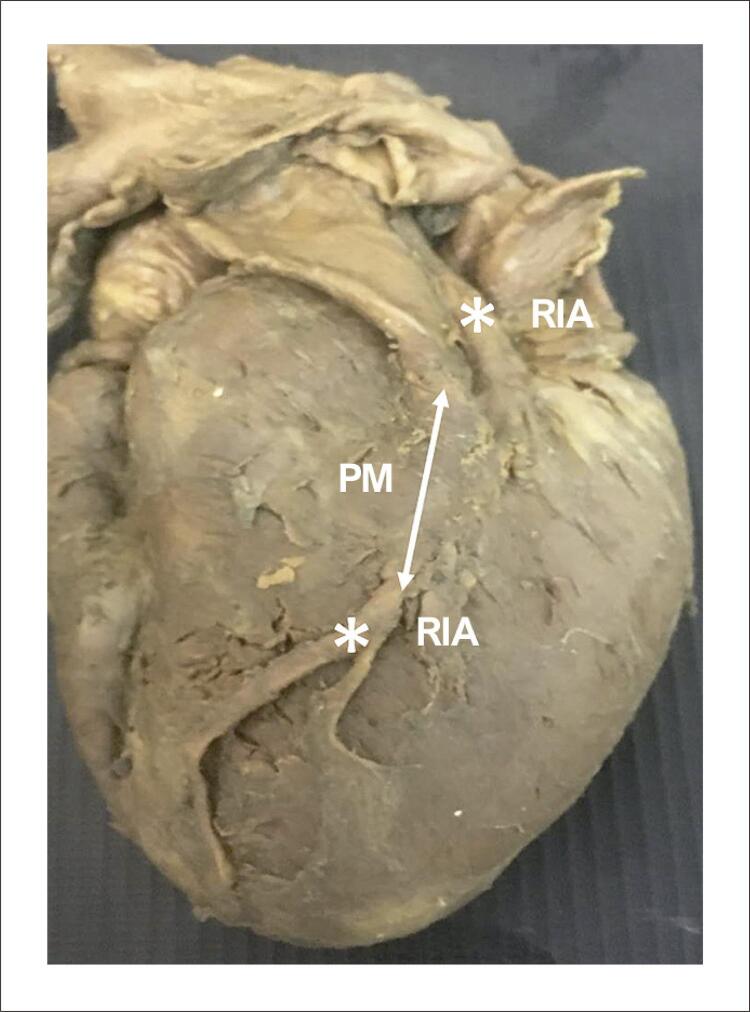
Identificação de uma ponte miocárdica (PM). PM: ponte miocárdica; RIA: ramo interventricular anterior.

O maior comprimento médio das pontes miocárdicas foi localizado acima do RIA, que foi de 10,76 ± 5,69 mm (variando de um mínimo de 3,97 mm a um máximo de 22,95 mm), e as duas pontes mais longas (19,92 mm e 22,95 mm, respectivamente) localizavam-se acima dessa artéria ( Figura 1 ). A maior espessura média ocorreu nas pontes localizadas acima do RIA, que totalizaram 1,0 ± 0,32 mm, com ampla faixa de mínimo de 0,54 mm a máximo de 1,83 mm, que incluiu a ponte mais espessa e uma das duas pontes mais finas ( Figura central ).

Neste estudo, o IMMPM da PM também apresentou o maior valor médio nas pontes localizadas acima do RIA em relação ao grupo das pontes localizadas em outros ramos (p=0,02) ( Tabela 3 , Figura central ).

**Tabela 3:** – Diferenças dos valores médios do IMMPM entre pontes localizadas acima do RIA e as localizadas em outros ramos

		N	Média	DP	p
IMMPM	RIA	16	11,18	8,18	0,02
	Outros ramos	11	3,83	1,75	

N: número de observações; RIA: ramo interventricular anterior; IMMPM: índice de massa muscular da ponte miocárdica; DP: desvio padrão.

## Discussão

O presente estudo obteve uma prevalência de PM (40%) semelhante a outro estudo realizado com população do nordeste do Brasil (40,4%).^17^ Entretanto, essa prevalência foi mais alta do que a encontrada em outros estudos: 1,44%,^18^ 3,9%,^11^ e 19%^6^ na literatura. Sabe-se que o diagnóstico de PM depende do método de avaliação. Uma revisão sistemática encontrou uma prevalência média de 1,9% para angiografia (n = 2.141 de 110.203 casos avaliados), 18,9% para angiotomografia (CCTA) (n = 8.313 casos de 43.904 avaliados) e 32,9% para autópsias (n = 1.442 em 4.384 casos).^5^ Portanto, o valor obtido é corroborado por estudos que observaram uma prevalência de aproximadamente 40%.^5 , 19 , 20^ Essa ampla discrepância nos valores de prevalência pode ser explicada pelos diferentes métodos utilizados para determinar o diagnóstico de PM e pela amostra avaliada.^5 , 18^ Apesar do contraste na prevalência da PM, exames de imagem, tais como angiografia,^2 , 5 , 9 , 11 , 14 , 21^ ultrassom intravascular,^2 , 18^ e CCTA,^2 , 5 , 8^ são usados para definir o diagnóstico in vivo.

Em quase metade dos casos, o diagnóstico só é possível por meio de autópsia, principalmente em alterações morfológicas menores que 200 micrômetros.^15^ Portanto, estudos post-mortem demonstram uma sensibilidade diagnóstica maior do que os métodos diagnósticos indiretos (exames de imagem)^2 , 5 , 7^ e um índice mais baixo de falsos negativos, devido ao acesso visual, possibilidade de dissecção, manipulação física, e menor impacto da técnica e experiência do avaliador. A PM é uma anomalia congênita com uma incidência relatada marcadamente variável na autópsia (4,7% – 86%), provavelmente relacionada a região geográfica. Esses dados foram coletados da autópsia realizada em 100 corações com registros médicos.^3^

A PM é um fator de risco de aterosclerose,^2 , 6 , 8 - 11 , 14 - 16 , 18 , 19 , 21 - 23^ especialmente em pacientes com diabetes mellitus.^8^ Essa relação é especialmente importante para considerar o risco de doenças cardiovasculares mais graves, tais como o IAM.^9 , 11 , 15 , 19^ Entretanto, o diagnóstico de PM apresenta um prognóstico considerado bom, com variações de 0 - 5% de mortalidade, sem IAM, em acompanhamentos de 2 - 5 anos.^11 , 19 , 21^ É importante ressaltar que a utilização desses exames complementares, como o ultrassom intravascular e a tomografia computadorizada cardíaca, tem aumentado a sensibilidade para detecção de PM in vivo, bem como possibilitando melhores caracterizações morfológicas e funcionais desses achados anatômicos em pacientes,^1 , 2 , 8^ permitindo diagnóstico precoce, tratamento e melhor qualidade de vida, mesmo em pacientes sem sintomas cardiovasculares.^9 , 17 , 19^

O comprimento da PM variou de 2,79 a 22,95 mm, com média de 8,55 mm. Esse padrão foi mais baixo do que o encontrado na literatura,^5 , 22^ com uma média de 19,3 mm em uma análise sistemática.^5^ É sabido que o comprimento é variável, mesmo quando se considera o método de avaliação,^5 , 23^ provavelmente devido às limitações da técnica. Para os métodos de angiografia e angiotomografia, foi relatado um comprimento médio de 21,0 mm.^5^ A espessura média foi de 0,89 mm, maior que os 0,46 mm obtidos em um estudo^23^ e menor que os achados de uma revisão sistemática (3,2 mm e 3 mm em autópsias e CCTA/angiografias, respectivamente).^5^

O IMMPM foi quase 3x mais alto no tronco interventricular anterior da ACE do que em outros troncos coronários. O IMMPM é calculado pelo produto do comprimento da PM e a espessura do halo (profundidade). Quando separados, comprimento^10 , 23^ e espessura^10^ estão relacionados à sintomatologia cardiovascular do indivíduo; entretanto, comprimentos ou espessuras grandes apresentam um risco mais alto de expressão de sintomas cardiovasculares.^10^ Além disso, no que diz respeito ao IMMPM, existe uma relação entre altos escores e disfunção hemodinâmica, mas esse índice pode fornecer informações não invasivas sobre o impacto das PM nos vasos afetados.^24^ Portanto, o RIA expressa um prognóstico clínico pior.

Os estudos demonstram que as PM que passam sobre o vaso sanguíneo poderiam ter uma grande capacidade de contração e, consequentemente, uma grande força de compressão, que seria exercida na parede de um vaso sanguíneo. Embora algumas PM possam ser assintomáticas, sua presença geralmente causa doenças coronárias, seja por compressão direta do segmento ou por simulação de um desenvolvimento acelerado de aterosclerose no segmento proximal à PM.^25^

Em relação ao desenho metodológico, embora o tamanho da amostra do estudo atual seja relativamente pequeno, é importante, já que apresenta informações que não estavam disponíveis anteriormente na literatura. Entretanto, é importante entender que a caracterização de amostra e viés de controle está presente devido à impossibilidade de se obter informações sobre essas características como idade, gênero, etnia, histórico clínico e causa da morte.

A ADAE, em seu tronco interventricular anterior (32%), ramo da ACE, foi o local mais afetado. Isso foi corroborado por um estudo brasileiro^17^ e parcialmente por uma revisão sistemática, demonstrando que a artéria interventricular anterior esquerda foi a mais afetada, mas com uma prevalência mais alta (79,3%).^5^ Os valores do ramo marginal esquerdo (8%) e do tronco ventricular posterior (2%) foram semelhantes (8,8% e 2,3%, respectivamente) aos encontrados na literatura.^5 , 26^ A prevalência do sistema coronariano direito (12% no RIP ou artéria descendente posterior direita) foi muito superior à dos achados anteriores (3,7%).^5^

## Conclusão

O presente estudo revelou um perfil de alta prevalência de PM, com alta probabilidade de estar presente no sistema coronariano esquerdo com índice de massa muscular maior que o encontrado em outros ramos acometidos, além de pior prognóstico. Portanto, para os clínicos, este estudo enfatiza a importância e a necessidade de uma investigação precoce da PM, mesmo em pacientes saudáveis sem sintomas de angina. Também preconiza a prevenção de eventos cardiovasculares importantes necessários para obter um melhor prognóstico e qualidade de vida para os pacientes.
