# Supplementary material for: Incidência e Estudo Morfológico de Pontes Miocárdicas no Estado do Ceará: Um Estudo Cadavérico
Source: Arq Bras Cardiol. 2023 Jun 28;120(7):e20220460. [Article in Portuguese] doi: 10.36660/abc.20220460 (PMC10365010; doi:10.36660/abc.20220460)
Supplement: Translation

# Translation: Incidence and Morphological Study of Myocardial Bridge in the State of Ceará: A Cadaveric Study

**Authors:** Jalles Dantas de Lucena, Hudson Martins de Brito, João Victor Souza Sanders, Jonathan Barros Cavalcante, Michelly Carneiro Collyer, Cecília de Lima Leite, Helson Freitas da Silveira, Julio Cesar Campos Ferreira

**Affiliations:** 1 Brazil Centro Universitário Santa Maria, Cajazeiras, PB – Brazil; 2 Brazil Universidade Federal do Ceará, Fortaleza, CE – Brazil

**Keywords:** Anatomy, Myocardial Bridging, Cardiovascular Abnormalities, Incidence

## Abstract

**Background:**

Myocardial bridges (MB) are anatomical anomalies with possible clinical repercussions; hence, their understanding deserves attention.

**Objective:**

To determinate the prevalence and characterize MB in human hearts from the state of Ceará. Methods: Fifty hearts of adult human cadavers from the Medicine School of Federal University of Ceará, Brazil. The hearts were dissected to identify MBs that pass over part of the coronary artery. The segment of the artery (proximal, middle, and distal) with a bridge was identified. The external diameter of the artery at the proximal and distal points of the MB was measured. The length and thickness of the MB were also measured with an electronic caliper. The muscle index (MMI) of the MB was calculated as the product of length and thickness expressed in millimeters. The significance level adopted in the statistical analysis was 5%.

**Results:**

MB was confirmed in 40% of sample. Approximately one third of the sample had only 1 MB. MB was most frequently found over the anterior interventricular branch of the left coronary artery (59.25%, p=0.02), and its prevalence in other branches was much lower (22.23%). The most affected segments of arteries were the superior (44.44%) and medium (40.74%). The mean diameter of arteries proximal to the MB was 2.38±0.97mm (range=0.78-5.15mm), and the diameter distal to the MB was 1.71±0.75mm (range=0.42-3.58mm). The length was measured as mean=8.55±5.27mm, while the mean thickness was 0.89±0.33mm.

**Conclusion:**

A high prevalence of MB is more likely to affect the left coronary artery system with larger MMI than other affected branches.

## Introduction

Myocardial bridge (MB) is a congenital coronary artery anomaly defined by the partial involvement of an arterial heart branch by myocardial muscle fibers, forming a muscle bridge over the involved vessels.^1 , 2^ MB can be found in any epicardial coronary artery; however, there is a greater prevalence along the course of the anterior interventricular branch (AIB) of the left coronary artery (LCA), also called left anterior descending coronary (LAD), in clinical studies.^3 , 4^ This anatomical variation is more common in the middle segment of the anterior descending coronary.^5^

A recent meta-analysis study showed a mean MB prevalence of around 19%.^6^ However, data on the frequency of MB are usually quite variable in the literature, depending on the methods used for their diagnosis. About this, the prevalence tends to be higher when considering post-mortem diagnoses.^2 , 7^ Currently, the use of new complementary exam techniques, such as intravascular ultrasound and cardiac computed tomography, has increased the sensitivity for MB detection, and has enabled a better morphological and functional characterization of these anatomical findings.^2 , 8^

The presence of MB is clinically relevant due to its association with anginal symptoms, myocardial ischemia, left-ventricular dysfunction, acute myocardial infarction (AMI), or even sudden death,^8 - 13^ and is also an important risk factor for coronary artery disease in various clinical situations.^8^ The pathophysiological mechanisms of ischemia associated with MB are still controversial and poorly understood due to the limitations of *in vivo* analysis.^2 , 8^ Once the diagnosis is established, the therapeutic measures are essentially drug-based and seek to maintain the heart rate at baseline values, which are considered first-line approaches.^14^

Stent implantation under the MB is, basically, a malpractice nowadays for a myriad of acute and long-term vital complications, while surgical decompression is debatable and offered in limited high-skilled centers; therefore, it is not feasible for all worldwide.^3^ In this perspective, it is necessary to investigate the prevalence of MB in populations, as it is a relatively common finding and an important risk factor for cardiovascular pathologies with high rates of morbidity and mortality, such as arteriosclerosis and AMI.^15 , 16^ Thus, this work aims to study the prevalence of MB in the branches of coronary arteries in dissected hearts in the state of Ceará in northeastern Brazil. Also analyze the length and thickness of the bridges, and whether there is a difference in the value of the muscle index of the MB located above the AIB and those bridges located above other branches of the coronary arteries.

## Material and methods

Fifty hearts of adult human cadavers were obtained for convenience from the Anatomy Department, School of Medicine, Federal University of Ceará, Ceará, Brazil. The number of hearts was not higher due to the scarcity of available specimens. The hearts were preserved in glycerin until analysis. Information, including age, gender, ethnicity, medical history, and cause of death could not be acquired due to the absence of records.

The specimens were selected from an intentional non-probabilistic sampling according to the following criteria: heart with the presence of the main arterial trunk and its branches preserved in subepicardial fatty tissue, preserved cardiac muscle, ensuring the visualization of the morphological characteristics of each specimen.

The epicardium and epicardial fat were carefully dissected. Thereafter, the origin and the course of the coronary arteries and their important branches were carefully delineated. All were followed carefully to see any bridging myocardium running over the arteries. If MB were detected, the segment of the artery (proximal, middle, and distal) with bridge was identified. The external diameter of the artery at the immediate proximal and distal points of the MB was measured. Subsequently, the length and thickness of the MB were also measured with the aid of a precise electronic caliper (DIGIMESS®, São Paulo, Brazil) of 0.01 mm accuracy. The muscle index of MB was calculated as the product of length and thickness expressed in millimeters.

### Statistical analysis

The data were collected in excel software sheets and analyzed statistically using GraphPad Prism, version 6.00, for Windows, California USA. Continuous variables were described through mean and standard deviation (SD) and were analyzed as normality by the Shapiro-Wilk test. The categorical variables were presented in tables, with the frequency of the studied variables. Comparisons between variables were performed using the unpaired Student’s *t* -test. Values at p < 0.05 were considered statistically significant. This anatomical study was performed after the agreement of the ethical committee of the S chool of Medicine, Federal University of Ceará, Ceará, Brazil.

## Results

The presence of a MB was confirmed in 40% of the hearts (n=20). Approximately one third of the sample (n=16, 32%) had only one MB. our study also found 4% (n=2) with two MBs (in different arteries) and 2% (n=1) with three MBs (2 MBs above the AIB) and four MBs (2 MBs above AIB) - Central Illustration .

**Central Illustration:** : Incidence and Morphological Study of Myocardial Bridge in the State of Ceará: A Cadaveric Study

Main findings in this article. MB: myocardial bridge; AIB: anterior interventricular branch; PIB: posterior interventricular branch; LMB: left marginal branch.

Twenty-seven MBs were identified in the sample, which were most commonly found above the AIB (n=16, 59.25%). Frequency of bridges above other branches was much lower, with 22.23% (n=6) above the posterior interventricular branch (PIB) and 14.82% (n=4) above the left marginal branch (LMB) ( Table 1 , Central Illustration ).

**Table 1:** – Distribution of Myocardial Bridges (n=27) above the arteries in the hearts (n=50)

Blood vessel	Hearts with MB %	Number of MB
**AIB**	32.0	16 (59.25)
**PIB**	12.0	6 (22.23)
**LMB**	8.0	4 (14.82)
**PVB**	2.0	1 (3.7)
**Total**	-	**27 (100.0)**

MB: myocardial bridge; AIB: anterior interventricular branch; PIB: posterior interventricular branch; LMB: left marginal branch; PVB: posterior ventricular branch.

The MBs were present mainly in the superior (44.44%) and medium (40.74%) segments of the arteries ( Central Illustration ). The mean diameter of arteries proximal to the MBs was 2.38±0.97 mm (range= 0.78-5.15 mm) and the diameter distal to the MBs was 1.71±0.75 mm (range = 0.42-3.58 mm).

The mean length of MBs amounted to 8.55±5.27 mm (range= 2.79-22.95 mm), while the mean thickness was 0.89±0.33 mm (range= 0.37-1.83 mm) - Central Illustration . Furthermore, the value of the Myocardial Bridge Muscle Index (MBMMI) ranged from a minimum of 1.70 mm to a maximum of 28.69 mm and amounted to 8.19±7.30 mm in the three arteries ( Table 2 ).

**Table 2:** – Morphological aspects of myocardial bridges (N=27)

	Length (mm)	Thickness (mm)	MBMMI
Mean	8.55	0.89	8.19
Std. deviation	5.27	0.33	7.30

MBMMI: Myocardial Bridge Muscle Index (length x thickness).

The greatest mean length of the MBs was located above the AIB, which amounted to 10.76±5.69 mm (ranging from a minimum of 3.97 mm to a maximum of 22.95 mm), and the two longest bridges (19.92 mm and 22.95 mm, respectively) were located above that artery ( Figure 1 ). The greatest average thickness occurred in the bridges located above the AIB, which amounted to 1.0±0.32 mm, with the broad range from a minimum of 0.54 mm to a maximum of 1.83 mm, which included the thickest bridge and one from the two thinnest bridges ( Central Illustration ).

**Figure 1:** – Identification of a myocardial bridge. MB: myocardial bridge; AIB: anterior interventricular branch.

In this study, the MBMMI of the MB also showed the highest mean value in bridges located above the AIB as compared to the group of bridges located on other branches (p=0.02) ( Table 3 , Central Illustration ).

**Table 3:** – Difference of the mean values of the MI between bridges located above the AIB and those located on other branches

		N	Mean	SD	p
MI	AIB	16	11.18	8.18	0.02
	Other branches	11	3.83	1.75	

N: number of observations; AIB: anterior interventricular branch; MI: muscle index of the myocardial bridge; SD: standard deviation.

## Discussion

The current study obtained a prevalence of MBs (40%) similar to another study conducted with a population in northeastern Brazil (40.4%).^17^ However, this prevalence was higher than other studies: 1.44%,^18^ 3.9%,^11^ and 19%^6^ reported in the literature. It is well-known that the diagnosis of MB depends on the method of assessment. A systematic review found a mean prevalence of 1.9% for angiography (n=2.141 out of 110.203 cases evaluated), 18.9% for angiotomography (CCTA) (n=8.313 cases out of 43.904 evaluated), and 32.9% for autopsies (n=1442 in 4.384 cases).^5^ Therefore, the obtained value is corroborated by studies that observed a prevalence of approximately 40%.^5 , 19 , 20^ This broad discrepancy in prevalence values can be explained by the different methods used to determine the diagnosis of MB and the evaluated sample.^5 , 18^ Despite the contrast in the prevalence of MB, imaging tests, such as angiography,^2 , 5 , 9 , 11 , 14 , 21^ intravascular ultrasound,^2 , 18^ and CCTA,^2 , 5 , 8^ are used to define the *in vivo* diagnosis.

In nearly half of the cases, the diagnosis is only possible by means of an autopsy, mainly in morphological alterations smaller than 200 micrometers.^15^ Therefore, post-mortem studies show a greater diagnostic sensitivity than do indirect diagnostic methods (imaging tests)^2 , 5 , 7^ and a lower rate of false negatives, due to visual access, possibility dissection, physical manipulation, and less impact of the technique and experience of the evaluator. The MB is a congenital anomaly with a markedly variable reported incidence on autopsy (4.7%–86%), likely related to geographic regions. This data was collected from the autopsy study performed on 100 hearts with medical records.^3^

MB is a risk factor for atherosclerosis,^2 , 6 , 8 - 11 , 14 - 16 , 18 , 19 , 21 - 23^ especially in patients with diabetes mellitus.^8^ This relationship is particularly important to consider the risk of more severe cardiovascular diseases, such as AMI.^9 , 11 , 15 , 19^ However, the diagnosis of MB presents a prognosis considered good, with variations of 0-5% of mortality, without AMI, in follow-ups of 2-5 years.^11 , 19 , 21^ It is noteworthy that the use of these complementary tests, such as intravascular ultrasound and cardiac computed tomography, has increased the sensitivity for detecting MB *in vivo* , as well as enabled better morphological and functional characterizations of these anatomical findings in patients,^1 , 2 , 8^ allowing for early diagnosis, treatment, and a better quality of life, even in patients without cardiovascular symptoms.^9 , 17 , 19^

The MB length ranged from 2.79 to 22.95 mm, with an average of 8.55 mm. This pattern was lower than in the literature,^5 , 22^ with a mean of 19.3 mm in a systematic review.^5^ It is well-known that the length is variable, even when considering the evaluation method,^5 , 23^ most likely due to the limitations of the technique. For the methods of angiography and angiotomography, a mean length of 21.0 mm was reported.^5^ The average thickness was 0.89 mm, which was greater than the 0.46 mm obtained in one study^23^ and less than the findings of one systematic review (3.2 mm and 3 mm in autopsies and CCTA/angiographies, respectively).^5^

The MBMMI was almost 3x higher in the anterior interventricular trunk of the LCA than in other coronary trunks. The MBMMI is calculated by the product of the MB length and halo thickness (depth). When set apart, length^10 , 23^ and thickness^10^ are related to the individual’s cardiovascular symptomatology, while high length or thickness present a greater risk of expressing cardiovascular symptoms.^10^ Furthermore, concerning MBMMI, there is a relationship between high scores and hemodynamic dysfunction, but this index may provide non-invasive insight into the impact of MBs on affected vessels.^24^ Therefore, AIB expresses a worse clinical prognosis.

Studies show that MBs that pass over the blood vessel could have a great contractive power and, consequently, a great compressive force, which would be exerted on the wall of a crossed blood vessel. Although some MBs can be asymptomatic, their presence often causes coronary disease, either through direct compression of the segment or through stimulation and accelerated development of atherosclerosis in the segment proximal to the MB.^25^

Regarding the methodological design, although the sample size of the current study is relatively small, it is important, as it presents information that was previously unavailable in the literature. However, it is important to understand that the sample characterization and control bias is present due to the impossibility of obtaining information on such characteristics as age, gender, ethnicity, clinical history, and cause of death.

The Left Anterior Descending Artery in its anterior interventricular trunk (32%), branch of the LCA, was the most affected location. This was corroborated by a Brazilian study^17^ and partially by a systematic review, showing that the left anterior interventricular artery was the most affected, but with a higher prevalence (79.3%).^5^ The LMB (8%) and posterior ventricular trunk (2%) were similar (8.8% and 2.3%, respectively) to the literature.^5 , 26^ The prevalence of the right coronary system (12% in the PIB or right posterior descending artery) was much higher than previous findings (3.7%).^5^

## Conclusion

The present study revealed a profile for the high prevalence of MB, with a high probability of being present in the left coronary artery system with a larger MMI than that found in other affected branches, as well as a worse prognosis. Therefore, for clinicians, this study emphasizes the importance and need for an early investigation of MB, even in healthy patients without anginal symptoms. It also calls for the prevention of important cardiovascular events needed to obtain a better prognosis and quality of life for patients.
